# Aeroallergen Sensitization Status in West China from 2024 to 2025

**DOI:** 10.3390/jcm15103644

**Published:** 2026-05-09

**Authors:** Siqi Guo, Zhengxiang Gao, Lingyi Yan, Yu Wu, Yu Gou, Leiwen Peng, Yifei Duan

**Affiliations:** 1 Department of Laboratory Medicine, West China Second University Hospital, Sichuan University, Chengdu 610041, China; tracy47@163.com (S.G.); gaozx84@163.com (Z.G.); yanlingyi95@163.com (L.Y.); wy18008278520@163.com (Y.W.); 13880787601@163.com (Y.G.); lwpeng99@163.com (L.P.); 2 Key Laboratory of Birth Defects and Related Diseases of Women and Children, Sichuan University, Ministry of Education, Chengdu 610041, China

**Keywords:** aeroallergen sensitization, aeroallergens, children, West China

## Abstract

**Background/Objectives**: There is an abundance of evidence to support the associations of allergic sensitization with asthma. Reducing exposure to allergens can help prevent the progression of asthma. Given the significant regional variations in allergen distribution patterns, our objective was to characterize the sensitization patterns of common childhood aeroallergens in a large, hospital-based pediatric cohort from West China through epidemiological surveys, thereby providing region-specific key data to guide the development of targeted prevention strategies and clinical management protocols. **Methods**: We analyzed data from 30,565 multiple allergosorbent tests collected by our hospital from 2024 to 2025. We additionally collected sex and age. If the level of an aeroallergen was 0.35 IU/mL or more, the test result for that aeroallergen was defined as positive, and positive cases were defined as those where one aeroallergen was positive. The positive rates for aeroallergens were calculated using the total number of tested children in each category (such as season, gender or age group). **Results**: A total of 30,565 children in West China were surveyed, with an overall allergen sIgE positive rate of 37.7%. Autumn had the highest positive rate (41.2%), which was significantly higher than those of the other seasons (*p* < 0.01). Indoor allergens had higher positive rates than outdoor ones. The top indoor allergens were *Dermatophagoides pteronyssinus* (30.18%), *Dermatophagoides farinae* (27.13%), and *house dust* (22.99%); the top outdoor allergens were *Cupressus* (10.22%), *Betula platyphylla* (5.01%), and *Sycamore* (4.24%). The positive rate increased with age: 18.49% in infants and toddlers, 38.58% in preschool children, and 45.96% in school-aged children (all *p* < 0.01). Males (39.76%) had a higher total positive rate than females (34.90%) (*p* < 0.01), with significant gender differences in five allergens, including *Dermatophagoides farinae*, *Dermatophagoides pteronyssinus*, *house dust*, *Alternaria*, and *Sycamore*. **Conclusions**: This study provides detailed epidemiological data on aeroallergen sensitization patterns among children, including seasonal, age-related, and sex-related differences. These findings may help inform region-specific preventive strategies and guide future research on allergen avoidance.

## 1. Introduction

Asthma is the most common chronic noncommunicable disease among children, characterized by chronic airway inflammation and hyperresponsiveness that can lead to recurrent coughing, wheezing, chest tightness, and shortness of breath. It represents a global health concern, affecting approximately 14% of children worldwide—a prevalence significantly higher than the 7.7% rate observed in adults [1]. In fact, over one-third of adults with asthma first developed the condition during childhood [2]. Allergic asthma is the most common phenotype in pediatric asthma, characterized by eosinophilic airway inflammation associated with sensitization to various allergens via specific immunoglobulin E (sIgE) antibodies, as confirmed by serological test or skin prick test.

Asthma exacerbations are the most common cause of asthma-related hospitalizations and can be life-threatening for children. Untreated exacerbations increase the risk of chronic obstructive pulmonary disease in adulthood. Asthma remains a major problem in China, with childhood prevalence increasing by over 50% every decade [3]. Although the incidence of asthma exacerbations has slightly decreased in recent years with increased focus on children’s health, the growing number of children with asthma continues to drive increases in asthma-related hospitalizations and deaths among children. Therefore, greater efforts are needed to reduce the incidence of asthma exacerbations in children. Sensitization to airborne allergens correlates with severe asthma and severe asthma exacerbations [4]. While allergen avoidance proves challenging in clinical practice, mounting evidence suggests tangible clinical benefits from reducing allergen exposure in sensitized patients when achievable. These clinical benefits include fewer days with persistent asthma symptoms, reduced hospitalizations, and decreased asthma-related unscheduled emergency or outpatient visits [5]. Thus, the timely identification of asthma patients and investigation of their potential sensitization to airborne allergens is particularly important.

In China, the burden of allergic diseases is especially severe, with over 45 million asthma cases and an estimated 200 million individuals suffering from allergic rhinitis, though a substantial proportion remain undiagnosed [6]. The pattern of allergen sensitization among Chinese individuals is highly complex, driven by significant regional variations in allergen distribution and population susceptibility [7]. Previous studies have reported that house dust mites are prevalent in southern and eastern China, while pollen allergies are common in western and northern regions [8]. The prevalence of dust mite allergy among patients with allergic diseases varies from 11.21% in Northeast China to 40.79% in South China. Dust mite allergy contributes to the development of allergic rhinitis and asthma [9]. A study conducted in the northern grasslands of China found that the most common types of pollen among pollen-induced allergic rhinitis patients were *Artemisia plants*, *Chenopodium plants*, and *climbing Humulus* [10]. In this study, we measured sIgE levels of 18 common allergens (including *Dermatophagoides pteronyssinus*, *House dust*, *Cat dander*, *Dog dander*, and others) in 30,565 children. These measurements provide a more accurate understanding of their sensitization to different allergens. The findings will reveal the primary allergens causing allergies in children in West China, which is crucial for developing targeted prevention and treatment strategies.

In summary, this study aims to describe the prevalence and patterns of aeroallergen sensitization in a hospital-based pediatric population from West China by measuring sIgE levels. These descriptive findings are intended to generate hypotheses and provide foundational data that may inform the design of future, targeted interventional studies on allergy prevention and asthma management in this region.

## 2. Materials and Methods

The data were retrospectively collected from pediatric outpatients who were treated at West China Second University Hospital from 1 August 2024 to 31 July 2025. Our hospital is a medical center in the western region of China, and its service area covers the entire western region. This study complies with the Declaration of Helsinki, and no personally identifiable information is included in the full text. It was approved by the West China Second University Hospital Ethical Committee (Approval no. 2023.407). The date when we collected the data was after the ethics approval was granted.

The age groups of the children were categorized as follows: infants and toddlers (<3 years), preschool children (3–<6 years), and school-age children (6–14 years) [11]. Data were sourced from hospital information systems, laboratory information systems, electronic medical records, and other databases. For subjects who underwent multiple tests during the study period, only the first test record was included.

The sIgE assay for pediatric patients used an enzyme-linked immunosorbent assay (HOB Biotech Group Corp., Ltd., Suzhou, Jiangsu, China), performed on a fully automated enzyme-linked immunosorbent analyzer, URANUS AE95 (Aikang MedTech Co., Ltd., Shenzhen, Guangdong, China). All pediatric patients provided 4 mL of venous blood collected in disposable vacuum tubes (Becton, Dickinson and Company, Franklin Lakes, NJ, USA). Samples were centrifuged at 3500 rpm for 5 min to separate serum, which was then tested for allergens using the ELISA capture method. The panel included 18 allergens: inhaled indoor allergens (*Dermatophagoides farinae*, *Dermatophagoides pteronyssinus*, *House Dust*, *Dog dander*, *Cat dander*, *Cockroach*, *Alternaria*, *Penicillium punctatus*, *Aspergillus fumigatus*) and inhalant outdoor allergens (*Humulus japonicus*, *Betula platyphylla*, *Sycamore*, *Cottonwood*, *Cupressus*, *Pine*, *Mugwort*, *Common ragweed*, *Willow*). Procedures followed the instrument and kit instructions. Prior to conducting the test, we verified the performance of both the kit and the instrument. This verification assessed parameters including the detection limit, precision, carryover contamination rate and clinical concordance, following the quality specifications of the International Organization for Standardization (ISO) 15189 [12] for medical laboratories. The laboratory also performs daily internal quality control with manufacturer-supplied materials. Furthermore, it participates in the external quality assessment scheme administered by the National Clinical Research Center in China. These measures ensure the accuracy of the results. Results were graded and interpreted according to international standards [13]. The standard diagnostic threshold for allergen sensitization is set at 0.35 IU/mL; if the level exceeds this threshold, it is considered that the individual is allergic to the allergen. If the level of an aeroallergen was 0.35 IU/mL or more, the test result for that aeroallergen was defined as positive, and positive cases were defined as those where one aeroallergen was positive.

Data processing was performed using GraphPad Prism 8.0 and SPSS software (v27). Count data were expressed as case numbers or percentages. The positive rates for aeroallergens were calculated using the total number of tested children in each category (such as season, gender or age group). Comparisons of rates were performed using the chi-square test or exact probability method, with *p* < 0.05 considered statistically significant. Given the exploratory and descriptive nature of our study, we did not apply a formal correction for multiple comparisons, such as the Bonferroni method. This approach was taken to minimize the risk of Type II errors. It also helped prevent the potential masking of meaningful patterns in the data.

## 3. Results

This retrospective analysis evaluated the allergen sensitization profiles of 30,565 pediatric patients (17,513, 57.30% male; 13,052, 42.70% female; male/female ratio 1.34:1) who presented to our hospital. Table 1 shows the descriptive statistics of the number of patients during the study period. In terms of age, 15,538 patients (50.84%) were school-age children. This was followed by preschool children, 7989 (26.14%); the least number of patients were infants and toddlers, 7038 (23.03%).

**Figure 1:**
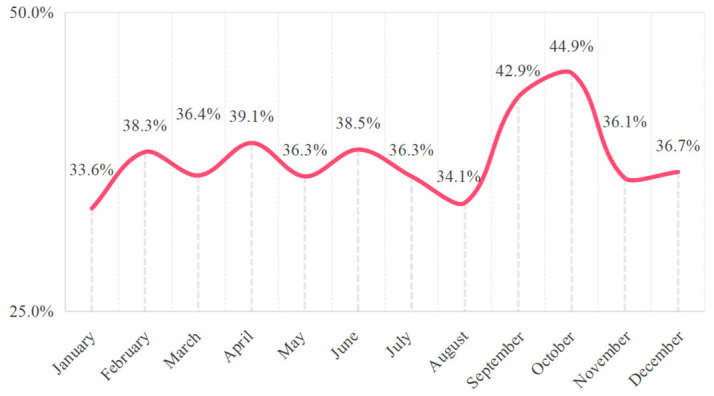
The monthly positive rate for sIgE. The positive rates for aeroallergens were calculated using the total number of tested children in each corresponding month.

The overall positive rate for sIgE to allergens in 30,565 children was 37.7% (11,524/30,565). The monthly positive rate was calculated using the total number of tested children in each corresponding month. It was shown in Figure 1. The spring positivity rate was 37.3% (3283/8797), the summer positivity rate was 36.2% (3086/8528), the autumn positivity rate was 41.2% (2878/6977), and the winter positivity rate was 36.4% (2277/6263). The difference in positivity rates between spring and summer was not statistically significant (RR = 1.015, 95% CI: 0.987–1.044; χ^2^ = 1.06, *p* = 0.29). The difference between spring and winter was not statistically significant (RR = 1.011, 95% CI: 0.985–1.037; χ^2^ = 0.67, *p* = 0.41). The difference between summer and winter was not statistically significant (RR = 0.998, 95% CI: 0.972–1.025; χ^2^ = 0.02, *p* = 0.88). Among the four seasons, autumn exhibited the highest positive rate. Comparisons between autumn and spring, summer, and winter all showed statistically significant differences (RR = 1.056, 95% CI: 1.023–1.090; χ^2^ = 11.03, *p* < 0.01; RR = 1.072, 95% CI: 1.039–1.107; χ^2^ = 18.41, *p* < 0.01; RR = 1.059, 95% CI: 1.029–1.091; χ^2^ = 14.64, *p* < 0.01).

**Figure 2:**
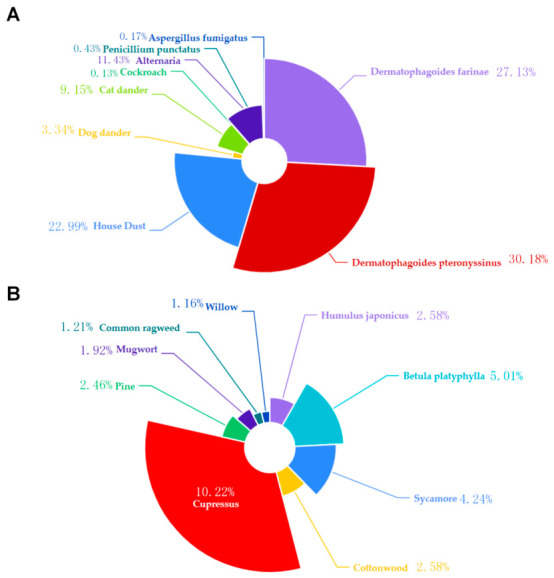
(**A**) showed the positive proportion of indoor allergens. The percentages were calculated based on the total number of tested children for each indoor allergens; (**B**) showed the positive proportion of outdoor allergens. The percentages were calculated based on the total number of tested children for each outdoor allergens.

Indoor allergens included *Dermatophagoides farinae*, *Dermatophagoides pteronyssinus*, *House dust*, *Dog dander*, *Cat dander*, *Cockroach*, *Alternaria*, *Penicillium punctatus*, and *Aspergillus fumigatus*, which showed positive rates of 27.13%, 30.18%, 22.99%, 3.34%, 9.15%, 0.13%, 11.43%, 0.43%, and 0.17%, respectively, among the total number of tested individuals. Among outdoor allergens, the positive rates for *Humulus japonicus*, *Betula platyphylla*, *Sycamore*, *Cottonwood*, *Cupressus*, *Pine*, *Mugwort*, *Common ragweed*, and *Willow* were 2.58%, 5.01%, 4.24%, 2.58%, 10.22%, 2.46%, 1.92%, 1.21%, and 1.16%, respectively. Among indoor allergens, the top three positive rates were *Dermatophagoides pteronyssinus* (*Dermatophagoides pteronyssinus* vs. *Dermatophagoides farinae*, RR = 1.053, 95% CI: 1.034–1.073; χ^2^ = 29.46, *p* < 0.01), *Dermatophagoides farinae* (*Dermatophagoides farinae* vs. *House dust*, RR = 1.083, 95% CI: 1.062–1.103; χ^2^ = 64.16, *p* < 0.01), and *House dust* (*House dust* vs. *Alternaria*, RR = 1.336, 95% CI: 1.312–1.360; χ^2^ = 776.2, *p* < 0.01) (Figure 2A). Among outdoor allergens, the top three positive rates were *Cupressus* (*Cupressus* vs. *Betula platyphylla*, RR = 1.342, 95% CI: 1.283–1.403; χ^2^ = 118.6, *p* < 0.01), *Betula platyphylla* (*Betula platyphylla* vs. *Sycamore*, RR = 1.083, 95% CI: 1.008–1.164; χ^2^ = 4.36, *p* < 0.05), and *Sycamore* (*Sycamore* vs. *Cottonwood*, RR = 1.243, 95% CI: 1.158–1.335; χ^2^ = 28.01, *p* < 0.01) (Figure 2B). The highest *Dermatophagoides pteronyssinus* positivity rate among indoor allergens was significantly higher than the highest *Cupressus* positivity rate among outdoor allergens, with a statistically significant difference between the two (RR = 1.183, 95% CI: 1.172–1.195; χ^2^ = 749.5, *p* < 0.01). Among indoor and outdoor allergens, *Dermatophagoides pteronyssinus* had the highest positive rate (*Dermatophagoides pteronyssinus* vs. *Dermatophagoides farinae*, RR = 1.053, 95% CI: 1.034–1.073; χ^2^ = 29.46, *p* < 0.01), while *Cockroach* and *Aspergillus fumigatus* had the lowest positive rates. The difference between the two was not statistically significant (RR = 1.219, 95% CI: 0.755–1.968; χ^2^ = 0.62, *p* = 0.43).

Comparison of total positive rates for allergen-sIgE among children of different age groups revealed that the total positive rate for infants and toddlers was 18.81% (1324/7038), for preschool children 40.28% (3218/7989), and for school-age children 48.51% (7537/15,538). Statistically significant differences in total allergen sIgE positivity rates across age groups were observed (χ^2^ = 857.4, *p* < 0.01). Specifically, the difference between infants/toddlers and preschool children was statistically significant (RR = 0.750, 95% CI: 0.733–0.769; χ^2^ = 445.8, *p* < 0.01). The difference in total positive rates between infants/toddlers and school-age children was statistically significant (RR = 0.809, 95% CI: 0.799–0.819; χ^2^ = 858.8, *p* < 0.01). The difference between preschool children and school-age children was also statistically significant (RR = 0.942, 95% CI: 0.928–0.957; χ^2^ = 54.6, *p* < 0.01). Distribution of allergens in different age groups is shown in Table 2. Among children of the same age group, the indoor allergen positivity rate for infants and toddlers was 17.59% (1238/7038), significantly higher than the outdoor allergen positivity rate of 1.22% (86/7038). Among preschool children, the indoor allergen positivity rate of 33.25% (2656/7989) was higher than the outdoor allergen positivity rate of 7.03% (562/7989). The indoor allergen positivity rate among school-age children (40.54%, 6299/15,538) was higher than the outdoor rate (7.97%, 1238/15,538), with statistically significant differences (infants and toddlers: RR = 7.698, 95% CI: 6.270–9.450; χ^2^ = 921.3, *p* < 0.01; preschool children: RR = 2.863, 95% CI: 2.652–3.091; χ^2^ = 1148.0, *p* < 0.01; school-age children: RR = 3.044, 95% CI: 2.889–3.207; χ^2^ = 2783.0, *p* < 0.01).

**Figure 3:**
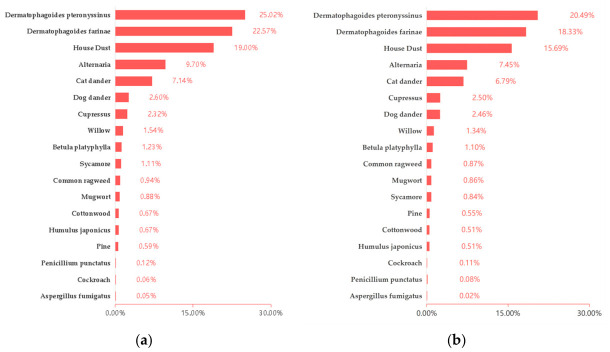
(**a**) Allergen positivity rates for males; (**b**) Allergen positivity rates for females. The positive rates for aeroallergens were calculated using the total number of tested children in each corresponding gender.

Comparing children of different genders, the overall positive rate for males was 39.76% (6964/17,513), significantly higher than the 34.90% (4555/13,052) observed in females (RR = 1.055, 95% CI: 1.037–1.074; χ^2^ = 34.31, *p* < 0.01). Allergen positivity rates for males and females were shown in Figure 3a and Figure 3b, respectively. For *Dermatophagoides farinae* (RR = 1.087, 95% CI: 1.064–1.111; χ^2^ = 53.92, *p* < 0.01), *Dermatophagoides pteronyssinus* (RR = 1.084, 95% CI: 1.062–1.106; χ^2^ = 54.23, *p* < 0.01), *House dust* (RR = 1.080, 95% CI: 1.056–1.106; χ^2^ = 39.83, *p* < 0.01), *Alternaria* (RR = 1.110, 95% CI: 1.077–1.144; χ^2^ = 40.12, *p* < 0.01), and *Sycamore* (RR = 1.114, 95% CI: 1.023–1.213; χ^2^ = 5.23, *p* < 0.05), the positive rates were significantly higher in males than in females. However, for *Willow* (RR = 1.057, 95% CI: 0.980–1.140; χ^2^ = 1.93, *p* = 0.16), *Common ragweed* (RR = 1.033, 95% CI: 0.937–1.140; χ^2^ = 0.41, *p* = 0.52), *Mugwort* (RR = 1.010, 95% CI: 0.912–1.120; χ^2^ = 0.04, *p* = 0.84), *Dog dander* (RR = 1.023, 95% CI: 0.964–1.086; χ^2^ = 0.55, *p* = 0.46), *Cat dander* (RR = 1.021, 95% CI: 0.984–1.060; χ^2^ = 1.22, *p* = 0.27), and *Cockroach* (RR = 0.698, 95% CI: 0.432–1.129; χ^2^ = 3.05, *p* = 0.08), *Humulus japonicus* (RR = 1.113, 95% CI: 0.998–1.241; χ^2^ = 3.16, *p* = 0.08), *Betula platyphylla* (RR = 1.047, 95% CI: 0.962–1.140; χ^2^ = 1.06, *p* = 0.30), *Cottonwood* (RR = 1.113, 95% CI: 0.998–1.241; χ^2^ = 3.16, *p* = 0.08), *Cupressus* (RR = 0.968, 95% CI: 0.907–1.034; χ^2^ = 0.98, *p* = 0.32), *Pine* (RR = 1.031, 95% CI: 0.912–1.167; χ^2^ = 0.23, *p* = 0.63), *Penicillium punctatus* (RR = 1.182, 95% CI: 0.927–1.508; χ^2^ = 1.38, *p* = 0.24), and *Aspergillus fumigatus* (RR = 1.309, 95% CI: 0.944–1.815; χ^2^ = 1.54, *p* = 0.22) showed no statistically significant differences in positive rates among children of different genders.

## 4. Discussion

The results of this study indicate that the overall sIgE positivity rate for allergens among 30,565 children in this research was 37.7%, with pronounced seasonal variation. The positivity rate peaked in autumn, significantly higher than in spring, summer, and winter, while no statistically significant differences were observed among spring, summer, and winter. The higher positivity rate observed in autumn is likely multifactorial. Although our study does not establish causality, we hypothesize that this may be related to regional climatic conditions favoring *house dust mite* proliferation and overlapping pollen seasons (e.g., *Mugwort*, *Common ragweed*) [14]. Furthermore, the differences in healthcare-seeking behavior or physician testing thresholds across seasons cannot be ruled out as contributing factors. These interpretations remain speculative and require confirmation through environmental monitoring and longitudinal studies. The combination of these factors may have increased the likelihood of children being exposed to allergens, thereby raising the positive rate of allergen tests.

The study identified the sensitization characteristics of indoor and outdoor allergens among children: indoor allergen positivity rates were generally higher than outdoor allergens, with *Dermatophagoides pteronyssinus*, *Dermatophagoides farinae*, and *House dust* emerging as core indoor allergens. The results were consistent with those from another hospital survey conducted in southern China. The sensitization to *house dust mites* (*D. pteronyssinus* and *D. farinae*) was the most common [15]. Among outdoor allergens, *Cupressus*, *Betula platyphylla*, and *Sycamore* ranked among the top three, and the positivity rate for the highest indoor allergens (*Dermatophagoides pteronyssinus*) was significantly higher than that for the highest outdoor allergens (*Cupressus*). This finding aligned with previous studies on sensitization patterns across different regions of China and also showed regional specificity: house dust mites, globally recognized as the primary indoor allergen, dominate both northern and southern China [16,17]. In contrast, the high positivity rates for pollen allergens such as Cupressus and *Betula platyphylla* are strongly correlated with the rich vegetation characteristic of western regions [18]. These results had identified key focus areas for our future research on indoor and outdoor allergens in this region.

Allergy prevalence rates show a significant upward trend across age groups: school-age children exhibit the highest overall positive rate, followed by preschool children and infants/toddlers, with statistically significant differences between all age cohorts. Furthermore, indoor allergen positivity rates exceed outdoor allergen rates across all age groups. This pattern may be linked to the developmental maturity of children’s immune systems, their activity ranges, and the cumulative effects of allergen exposure, although these mechanisms were not directly tested in our study. Infants and toddlers have underdeveloped immune systems and primarily remain in the home environment, which could contribute to lower sensitization rates [19]. As children progress from preschool to school age, their activities expand across multiple settings, potentially increasing exposure to diverse allergens. Concurrently, their immune systems mature, enhancing specific immune responses to allergens and progressively raising sensitization detection rates. The elevated sensitization rate among school-age children indicated this age group was a high-risk period for allergy-related diseases (such as asthma and allergic rhinitis). Clinicians should intensify screening efforts for this population to achieve early detection and intervention. Our results demonstrated the sensitization status of different age groups, providing a basis for the screening of high-risk populations with allergies.

This study found that male children had a significantly higher overall positive rate for allergen-specific IgE than females. Specifically, males had higher positive rates than females for five allergens: *Dermatophagoides farinae*, *Dermatophagoides pteronyssinus*, *house dust*, *Alternaria*, and *Sycamore*. However, no statistically significant gender differences were observed for the remaining 13 allergens. From a physiological perspective, estrogen may promote the production of IgE, while androgens may have an inhibitory effect, which may increase women’s susceptibility to certain allergens [20]. From a behavioral perspective, boys typically engage in outdoor activities more frequently and intensely than girls [21], thereby increasing their exposure to outdoor allergens. Although the overall positive rate of sIgE in boys was significantly higher than that in girls, our data could not determine the underlying mechanism. Behavioral differences (such as outdoor activity patterns) may also play a role, but this remains speculative. Further research is needed to distinguish between biological factors and environmental factors.

Understanding children’s patterns of sensitization to specific allergens allows identification of high-risk populations and informs targeted interventions to minimize exposure. Accumulating evidence from longitudinal cohort studies suggests that rigorously applied multimodal environmental control measures are associated with delayed onset and reduced severity of allergic disease in susceptible children. Our research has revealed specific patterns of aeroallergen sensitization among children of different ages and sexes in this region. These descriptive findings may help generate hypotheses for future environmental and interventional studies, but they do not directly inform prevention or control strategies for asthma.

Our results have pointed out the direction for future clinical and public health studies on the management of childhood allergies. Given that the peak period of allergic reactions occurs in autumn, it is possible to investigate whether the occurrence of allergies can be reduced by strengthening indoor environmental control (such as dehumidification, hot water cleaning of bedding, and using dust-proof covers) before and during this season [22,23]. And should clinicians maintain a low threshold for sIgE testing in children presenting with cough or wheezing in autumn? Is it necessary to implement age-based preventive measures, such as infants and toddlers benefit from home-focused *dust mite* reduction, while preschool and school-age children require additional attention to collective settings such as schools and kindergartens (regular cleaning, ventilation, hygiene education)? The positive rate of sIgE for boys was higher, and they may require earlier and more frequent screening [24]. From a public health perspective, can urban greening policies reduce planting of highly allergenic trees (*Cupressus*, *Betula platyphylla*, *Sycamore*) in areas with high pediatric allergy prevalence, thereby reducing the exposure to allergens [25]?

When interpreting these findings, several limitations warrant careful consideration: (1) We evaluated only the 18 most common respiratory allergens, excluding other clinically relevant pediatric allergens. Furthermore, since the antigens used in the testing were crude extracts, the possibility of cross-reactivity cannot be completely ruled out. (2) The single-center, hospital-based nature of the study means our sample consists of children who sought medical care; most participants sought medical care because they were experiencing allergic symptoms, which may have led to an overestimation of allergic reactions compared to the actual prevalence in the general population of this region. The findings may not be directly generalizable to the general pediatric population of West China. (3) Although we reported the basic demographic variables of age and gender, we acknowledge that the lack of more comprehensive clinical and demographic data is a major limitation. Information such as detailed clinical history, lifestyle habits, specific geographic origin within western China, and socioeconomic status was not systematically collected. The absence of these variables limits our ability to conduct a more detailed analysis of risk factors associated with sensitization patterns and restricts a comprehensive description of the clinical background of the sensitized population. In future epidemiological studies, we will adopt a standardized, prospective data collection process to incorporate these additional parameters. (4) A positive sIgE result indicates immunological sensitization, not a definitive clinical allergy diagnosis, and this distinction is critical for proper interpretation of our findings. This fundamental premise limits the scope of application of these epidemiological data in clinical decision-making or preventive health strategies. (5) No multivariable adjustment was performed given the descriptive scope of the study, and all reported associations were crude and descriptive; they have not been adjusted for potential confounders and should not be interpreted as independent or causal associations. Some statistical comparisons involved non-independent observations (e.g., the same child could be sensitized to multiple allergens), which we now acknowledge as a limitation of allergen-level analysis. Furthermore, and last but not least, to avoid Type II errors (i.e., false-negative results), this study did not perform multiple corrections; this decision may increase the risk of false-positive results. Therefore, future studies should employ more accurate statistical methods.

## 5. Conclusions

Our research indicates that indoor *Dermatophagoides pteronyssinus* and *Dermatophagoides farinae* are the main sources of allergens in our region, but the outdoor *Cupressus* should not be overlooked either. Especially in autumn, this is the season with the highest positive rate. The observed sensitization situations show significant differences among different ages and genders. It suggests that school-age boys may be a high-risk group. These findings provide valuable insights into the current status of respiratory allergen sensitization in the study area, although the biological basis for these observed differences still needs to be clarified through future research. At the same time, this study provides insights into potential directions for our future research, such as what measures should be taken during different seasons, how to determine screening frequencies for different ages and genders, as well as the role of schools and government greening departments in allergy prevention, and so on.

## Figures and Tables

**Table 1:** Characteristics of participants.

	Number of Participants	Percentage (%)
Sex		
Male	17,513	57.30%
Female	13,052	42.70%
Age group		
infants and toddlers (<3 years)	7038	23.03%
preschool children (3–<6 years)	7989	26.14%
school-age children (6–14 years)	15,538	50.84%
Month		
January	1841	6.02%
February	2196	7.18%
March	2868	9.38%
April	3157	10.33%
May	2772	9.07%
June	2644	8.65%
July	2797	9.15%
August	3087	10.10%
September	2141	7.00%
October	2396	7.84%
November	2440	7.98%
December	2226	7.28%
Total	30,565	

**Table 2:** Distribution of aeroallergen sensitization by age group.

Allergen	Age Group [*n* (%)]		
	Infants and Toddlers (*n* = 7038)	Preschool Children (*n* = 7989)	School-Age Children (*n* = 15,538)
Indoor allergens			
*Dermatophagoides pteronyssinus*	509 (7.23%)	1812 (22.68%)	4736 (30.48%)
*Dermatophagoides farinae*	395 (5.61%)	1579 (19.76%)	4370 (28.12%)
*House Dust*	625 (8.88%)	1339 (16.76%)	3411 (21.95%)
*Alternaria*	287 (4.08%)	746 (9.34%)	1638 (10.54%)
*Cat dander*	170 (2.42%)	397 (4.97%)	1569 (10.10%)
*Dog dander*	89 (1.26%)	170 (2.13%)	517 (3.33%)
*Penicillium punctatus*	1 (0.01%)	9 (0.11%)	21 (0.14%)
*Cockroach*	4 (0.06%)	2 (0.03%)	19 (0.12%)
*Aspergillus fumigatus*	0 (0.00%)	5 (0.06%)	7 (0.05%)
Outdoor allergens			
*Cupressus*	14 (0.20%)	240 (3.00%)	478 (3.08%)
*Willow*	16 (0.23%)	103 (1.29%)	325 (2.09%)
*Betula platyphylla*	20 (0.28%)	122 (1.53%)	217 (1.40%)
*Sycamore*	24 (0.34%)	118 (1.48%)	162 (1.04%)
*Common ragweed*	12 (0.17%)	69 (0.86%)	196 (1.26%)
*Mugwort*	20 (0.28%)	78 (0.98%)	168 (1.08%)
*Cottonwood*	11 (0.16%)	65 (0.81%)	109 (0.70%)
*Humulus japonicus*	6 (0.09%)	55 (0.69%)	124 (0.80%)
*Pine*	11 (0.16%)	71 (0.89%)	94 (0.60%)

Data are presented as *n* (%). Percentages are calculated using the total number of tested children within each age group as the denominator (infants and toddlers, *n* = 7038; preschool children, *n* = 7989; school-age children, *n* = 15,538).

## Data Availability

The datasets used and/or analyzed during the current study are available from the corresponding author upon reasonable request.

## Abbreviation

The following abbreviation is used in this manuscript:

sIgE	specific immunoglobulin E

## References

[1] Zheng J., Jin Y.J., Wang C.H., Feng C., Lai X.Y., Hua S.Q., Tai J.H. (2025). Global, regional, and national epidemiology of allergic diseases in children from 1990 to 2021: Findings from the Global Burden of Disease Study 2021. BMC Pulm. Med..

[2] Shin Y.H., Hwang J., Kwon R., Lee S.W., Kim M.S., Shin J.I., Yon D.K., GBD2019 Allergic Disorders Collaborators (2023). Global, regional, and national burden of allergic disorders and their risk factors in 204 countries and territories, from 1990 to 2019: A systematic analysis for the global burden of disease study 2019. Allergy.

[3] Tao M., Zhang Y., Ding L., Peng D. (2024). Risk factors of sleep-disordered breathing and poor asthma control in children with asthma. BMC Pediatr..

[4] Fouka E., Domvri K., Gkakou F., Alevizaki M., Steiropoulos P., Papakosta D., Porpodis K. (2022). Recent insights in the role of biomarkers in severe asthma management. Front. Med..

[5] Thao N.T.V., Kien T.G., Tuan T.A., Duc N.M., Hoang P.M., Vu L.T. (2023). Indoor Aeroallergen Sensitization and Associated Factors in Hospitalized Children with Asthma Exacerbations. Med. Arch..

[6] Zhen S., Yin Z., Duan J., Li Q., Pei H., Wu Q., Li Q., Zhang Y., Zhang H., Chen N. (2025). Pollen exposure and mild allergy: A multi-city study in China. J. Hazard. Mater..

[7] Yang F., Zhao X., Liu W., Zhou B., Deng L., Chen H., Zhang Z., Zhou L. (2023). Positive rate of wheat allergens in the Chinese allergic population: A systematic review and meta-analysis. Sci. Rep..

[8] Yuan X.W., Huang B., Feng G.R., Ye H.L., Chen P., Yao J. (2025). Prevalence of food and inhalant allergies in infants and children from the Nanhai area of Foshan city. BMC Pediatr..

[9] Liu S., Zhou Q., Dai B., Chen L., Zhang Q., Han L., Li X., Shen W., Shan L. (2024). Parents’ knowledge, attitudes and practices towards the prevention and treatment of dust mite allergy: A cross-sectional study in Shenyang (China). BMJ Open.

[10] Luo W., Li Y., Xu L., Yu Y., Ma J., Wang Y., Wang Y., Wu H., Xv M., Wu L. (2023). Pollen allergens sensitization characteristics and risk factors among allergy rhinitis of children in mainland China: A multicenter study. Heliyon.

[11] Huang S., Zhuang W., Xu T., Li Q., Wu L., Li J. (2025). Changes in *Mycoplasma pneumoniae* epidemiological and clinical features in children before, during, and after the COVID-19 pandemic in Shanghai, China. Microbiol. Spectr..

[12] (2022). Medical Laboratories—Requirements for Quality and Competence.

[13] Clinical Laboratory Standards Institute (2016). Analytical Performance Characteristics, Quality Assurance, and Clinical Utility of Immunological Assays for Human Immunoglobulin E Antibodies of Defined Allergen Specificities.

[14] Sun X., Li X., Gao T., Liu P., Liu N., Jin P., Zhi L. (2025). Reduction in Artemisia Pollen-Specific IgE Levels During House Dust Mite Allergen Immunotherapy in Polysensitized Allergic Rhinitis Patients: A Three-Year Retrospective Study in Northern, China. J. Asthma Allergy.

[15] Yang C., Zeng Q., Tang X., Li J., Liu W. (2024). Aeroallergen sensitization patterns in children with allergic rhinitis from Guangzhou and Liuzhou in southern China. AIMS Allergy Immunol..

[16] Chen H., Gong G.Q., Ding M., Dong X., Sun Y.L., Wan L., Gao Y.D. (2022). Dropouts from Sublingual Immunotherapy and the Transition to Subcutaneous Immunotherapy in House Dust Mite-Sensitized Allergic Rhinitis Patients. Front. Allergy.

[17] Hou Y.B., Sun J.L. (2024). Common pollen and related allergen components in patients with allergic diseases in the Beijing area. Front. Allergy.

[18] Ning X., Kuang Y., Zhao S., Hou W., Yang G., Zhu X., Liu R., Huang J. (2020). Design of an Optimally-Diagnostic Skin Test for Diagnosis of Sensitivity to Eight Allergens: A First-in-Human Study of Dose Escalation and Simultaneous Administration in Chinese Subjects. J. Asthma Allergy.

[19] Silva C.G., Luz V.F., Nunes V.L., Verzoto A.B.M., Cotrim A.C.M., Dos Santos W.B., França E.L., Honorio-França A.C. (2025). Colostrum-Derived Melatonin Plus PEG Microspheres Modulate the Oxidative Metabolism of Human Colostrum Phagocytes. Metabolites.

[20] Grafanaki K., Antonatos C., Maniatis A., Petropoulou A., Vryzaki E., Vasilopoulos Y., Georgiou S., Gregoriou S. (2023). Intrinsic Effects of Exposome in Atopic Dermatitis: Genomics, Epigenomics and Regulatory Layers. J. Clin. Med..

[21] Xu W., Zheng C., Fu C., Gong X., Fang Q., Yin Z. (2024). Epidemiological characteristics and prediction model construction of hand, foot and mouth disease in Quzhou City, China, 2005–2023. Front. Public Health.

[22] Dou M., Wang X., Li Y., Song J., Gong A. (2025). Occupational hazard exposures among archivists. Front. Public Health.

[23] Sözener Z.Ç., Öztürk B.Ö., Aydın Ö., Demirel Y.S., Pınar N.M., Bavbek S., Sin B.A., Mungan D. (2021). Coincidence of pollen season and coronavirus disease 2019 pandemic: Less time outdoors-lesser allergy symptoms in 2020. Asia Pac. Allergy.

[24] Zhu F.F., Chen J., Jia H.J., Xu H.S., Jiang T., Yang L.L. (2025). Analysis of serum allergen specific IgE detection results in children with allergic diseases in Hangzhou area. Zhonghua Yu Fang Yi Xue Za Zhi.

[25] Zuberbier T., Stevanovic K., Ansotegui I.J., Anto J.M., Bergmann K.C., D’Amato G., Grüntuch-Ernst A., Haahtela T., Maurer M., Pietikäinen S. (2024). Green Roof Gardens-Selecting Allergy-Friendly Vegetation: A Global Allergy and Asthma Excellence Network (GA^2^LEN) Position Paper. J. Allergy Clin. Immunol. Pract..

